# Design and Implementation of a Hypothermic Machine Perfusion Device for Clinical Preservation of Isolated Organs

**DOI:** 10.3390/s17061256

**Published:** 2017-06-01

**Authors:** Fei Shen, Ruqiang Yan

**Affiliations:** School of Instrument Science and Engineering, Southeast University, Nanjing 210096, China; mjz2861@163.com

**Keywords:** HMP device, physiology parameters monitoring, modified Bayes estimation, fuzzy-PID controller

## Abstract

The imbalance between limited organ supply and huge potential need has hindered the development of organ-graft techniques. In this paper a low-cost hypothermic machine perfusion (HMP) device is designed and implemented to maintain suitable preservation surroundings and extend the survival life of isolated organs. Four necessary elements (the machine perfusion, the physiological parameter monitoring, the thermostatic control and the oxygenation apparatus) involved in this HMP device are introduced. Especially during the thermostatic control process, a modified Bayes estimation, which introduces the concept of improvement factor, is realized to recognize and reduce the possible measurement errors resulting from sensor faults and noise interference. Also, a fuzzy-PID controller contributes to improve the accuracy and reduces the computational load using the DSP. Our experiments indicate that the reliability of the instrument meets the design requirements, thus being appealing for potential clinical preservation applications.

## 1. Introduction

The advent of clinical organ transplantation has attracted researchers to the task of preserving organs outside of the body, and proved to be the only way to treat several terminal stage diseases thoroughly [1]. However, the imbalance between the limited supply and huge potential need hinders the universal use of this technique. The three main preservation techniques for isolated organs include static cold storage (SCS), normothermic machine perfusion (NMP) and hypothermic machine perfusion (HMP). A general comparison between their performances is presented in Table 1. It indicates that organs preserved using SCS have a higher overall risk of transplant failure and shorter storage time compared with the machine perfusion strategy. Although the most expensive option, HMP mode combines the cooling protection advantage of SCS and the energy supply advantage of NMP. Since the conceptual basis of hypothermic machine perfusion came into being due to the early contributions of the Humphries team in the 1970s, many researchers have advanced this technique and applied it to cryopreserve kidneys, hearts, pancreatic islets and many other tissues [2,3]. However, compared to the SCS method, which is now in common use all over the world, the HMP technique is still not adopted by most transplantation centers due to its complexity and high cost [4,5], and was even given up in clinical care for a long period from the 1980s to the 1990s. On the other hand, researchers have proved that the machine perfusion style can effectively weaken the effects of ischemia and anoxia, resulting from the excision of tissue for transplantation, and its main anticipated and emerging benefits including maintaining the latency of the vascular bed [6], providing nutrients and low demand oxygen [6], removing metabolic by-products and toxins [7], providing access for cytoprotective or immunomodulatory drugs.

The basic principle of cell preservation is to minimize the deleterious effects of ischemia and anoxia while the organ is outside the body. Two main measures are used for the realization of this goal: low temperature and energy supply. Firstly, the benefit of cold on an abscised organ is predictable. When heat is removed from a biological system or a cell, the molecular activity or the chemical processes inside will slow down sharply, especially those degradation reactions related to ischemia and hypoxia: (1) slowing metabolism and demand for oxygen; (2) reducing the rate of substrate and energy depletion; (3) attenuating chemical processes that cause ischemic injury. However, freezing damage will occur in cells if the temperature is below a certain level, which will aggravate the organ deterioration. The negative influences include the dislocation of integrated biochemical pathways and ion transport and cell swelling, the membrane phase changes and the loss of phospholipids, cell apoptosis or necrosis, etc. According to [12] 4 °C is a suitable temperature level, and in that case, both ischemia and freezing damage are the lowest. Secondly, steadily providing energy to an organ on the brink of death will effectively extend its life. Reference [13] painted the basic trend of these physical parameters, including flow, resistance and temperature, measured on the LifePort Transporter [9] during 24 h hypothermic perfusion of a porcine pancreas [14]. It shows a continual increase of flow rate as well as the decrease of flow resistance during perfusion time in this system. However, although energy supply has remarkable benefits, excessive circulation will lead to a weight gain of organs and bring a potential risk of edema, due to the low handing ability of dying cells [11]. Therefore, a reasonable balance of both circulation speed and temperature should be controlled in the HMP device.

We notice that three main factors are considered in a classical HMP system: (1) the physical parameter control; (2) the composition of perfused solutions; and (3) the oxygen supply. However, two non-ignorable shortcomings exist in current HMP systems. Firstly, the exchangeability between different organs such as kidney and liver is poor because most organ saving boxes are designed directly for a specific organ. Secondly, although these parameters in current HMP devices can be monitored, the values need to be noted down by hand, which increases the risk of uncontrollable deviations from setting parameters.

It is our intent in this article to present a design of an inexpensive hypothermic machine perfusion system. The main design strategy is to wash out metabolic waste and replenish material energy using mechanical devices, such as compressors and peristaltic pumps. Meanwhile, to maintain a suitable organ living surrounding, the necessary physiological parameters in the designed HMP system are monitored in real-time, including the temperature of the storage space, the pH level of solutions, the perfusion pressure and the flow resistance. Compared with classical HMP devices, several remarkable advantages can be achieved: automation, multiple parameter monitoring and low application cost. In particular, the flow of artery solution in our equipment is pulsed with a human heart rate, which imitates the blood flow law and offers a more human-like organ living surrounding, aiming to extend the survival time of excised organs. Also, the modified Bayes estimation and Fuzzy-PID will be adopted to improve the thermal control precision of current HMP devices.

## 2. Primary Design Strategy and Materials

The current resurgence of interest in the clinical use of HMP was driven by reports in the early 1990s that preservation by machine perfusion reduces delayed function rates and increases the rate of prompt function of renal grafts [15,16]. Since then, some HMPs have been developed, such as the LifeCradle^TM^ HR system [17] for heart organs, the Groningen Machine Perfusion [18] for liver organs, the LifePort system [19] for kidney and pancreas organs.

**Figure 1:**
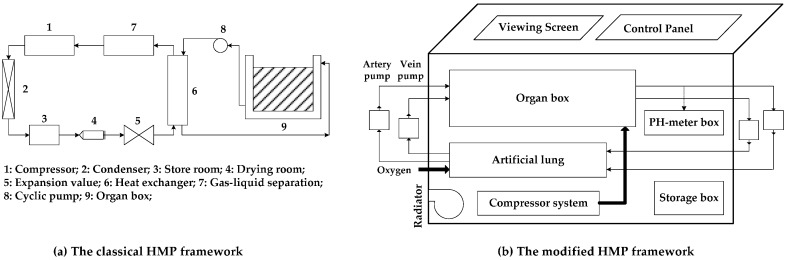
Two HMP frameworks. (**a**) In the classical HMP framework, the organ is saved in an isolated organ box and it uses the single compressor for temperature control; (**b**) In the modified HMP framework, oxygenated solution travels in the artery loop and the vein loop. Two peristaltic entrance pumps are used to promote the circular flow. Sensors are distributed in different parts for organ status monitoring.

**Figure 2:**
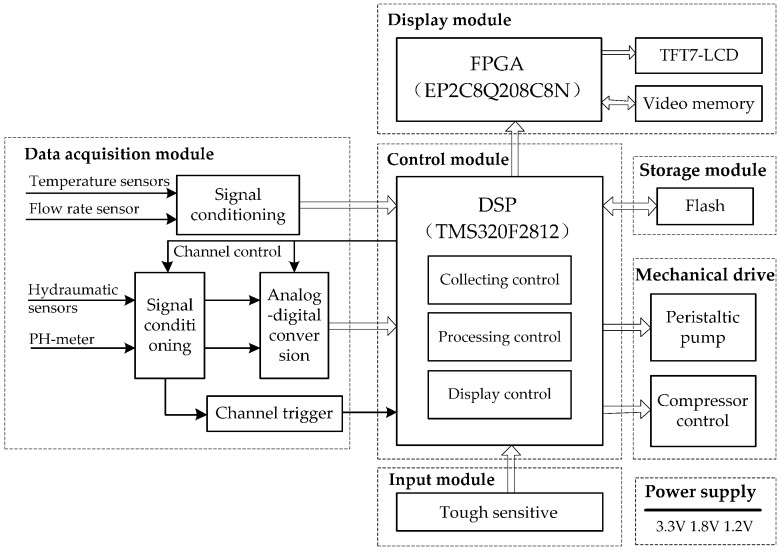
The circuit diagram of the HMP system. It contains a data acquisition module, a control module, a storage module, a mechanical drive, an input and display module, and a power supply.

The general structure of a classical HMP system, which mainly pay attention to the cooling control and ignore the influence of other parameters, is shown in Figure 1a. Therefore, a modified HMP system, shown in Figure 1b, was designed in this research. The advantage of the latter is that the range of monitored parameters can be adjusted according to the organ type, thus being more universal:

(a) Hardware circuit: during the design of the HMP device, a TMS320F2812 Digital Signal Processing (DSP) unit was considered as the microcontroller due to its strong performance in the industrial control field. Meanwhile, an EP2C8Q208 Field Programmable Gate Array (FPGA) was also employed for a 7 inches LCD monitor. Figure 2 presents the total circuit diagram of the HMP system, which includes the data acquisition sub-module, mechanical drive and LCD display;
(b) Physical properties of the perfusion solution: the perfusion solution is designed to be similar to human blood, where four main characteristics are noted: (1) a low viscosity for smooth flow in a 4–20 °C environment; (2) inert to reactions in a 2–8 °C environment; (3) a pH range is between 7.35 and 7.45;
(c) Environment requirements: to meet the medical field needs, some special requirements are considered: (1) the non-contact monitoring to avoid any pollution of the organs; (2) simplicity and convenience to replace a new organ; (3) flexible power supply.

## 3. Monitoring of the HMP System

### 3.1. Temperature Monitoring

Although cooling has proved to be the foundation of nearly all effective methods of protecting tissues for organ transplantation, too low a temperature may damage the cells and intensify the degradation process of organs outside the body [17]. The relatively optimum temperature for organ preservation is approximately 4 °C [20]. In that case, organs will achieve a good balance between hypothermic protection and cooling injury.

For temperature monitoring, four TSic506 [21] sensors are installed in the organ box in Figure 2 aiming to lower the risk of system failure, among which two of them are near the hot end and the other two sensors are close to the cold end. Then, a data fusion approach is implemented to improve the monitoring reliability. Here, the TSic506 unit is utilized due to its high precision (±0.1 °C) and suitable measurement range (−10~60 °C). The in-out relation of this kind of sensor is expressed as:

(1) $$T=\left[D_{T}\times \left(HT-LT\right)+LT\right]/2047\left(℃\right),$$

where T is the test point in degrees centigrade; $D_{T}$ is the output value of the TSic506; HT = 60 and LT = 10 are the upper and lower limits.

**Figure 3:**
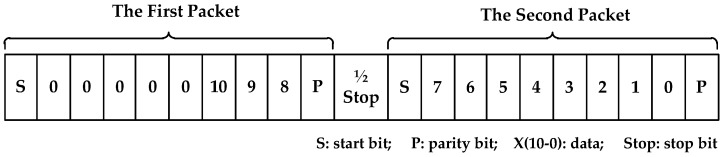
The frame format of Tsic506, (1) the first packet: 1 start bit, 3 data bits, 1 parity bit; (2) the second packet: 1 start bit, 8 data bits, 1 parity bit; (3) between the first packet and the second packet: 1/2 stop bit.

When the temperature is read from the Tsic506 sensor, it should be noticed that the $D_{T}$ value is stored in two units and they will be translated using two packets, which are contained in a whole frame format, as shown in Figure 3. The reading of $D_{T}$ value will be separated into the next three steps:

- Obtain the count period: the falling edge of the start bit triggers the count operation and stops when a new rising edge arrives. The count period $T_{s}$ represents the holding time of each bit accurately;
- Read translated packets: the DSP program waits a $T_{s}$ period after each falling edge, and then reads it. Each packet includes 8 bit data and 1 checksum;
- Data verification: inspect the checksum of two packets. If it is wrong, the program will return a mismark.

Although the monitoring accuracy of the Tisc506 meets the requirements of a hypothermic machine perfusion system, it is still far from enough. It is difficult to maintain exact temperature stability due to the inertia and time-lag of heat-spread in the perfusion fluid. We will introduce appropriate data fusion and thermostatic control approaches in the next section according to the temperature monitoring results.

### 3.2. Machine Perfusion Monitoring

As can be seen, machine perfusion is shown to be preferable for prolonged organ preservation times. The stronger pressure promotes the flow, thus retarding the accumulation of metabolic waste inside the organ, and then the deterioration of organ function can be limited, so the pressure and flow speed are two key parameters for machine perfusion.

Firstly, the flow resistance inside the blood vessel includes the straight pipe-resistance [22] and local resistance [23]. The former arises from the glutinousness of liquid as well as the friction between blood vessels and solution, and the latter results from the effect of crooks, bifurcations or confluences inside:

(2) $$h_{f}=\sum h_{z}+\sum h_{j}=\Delta p/\rho ,$$

(3) $$h_{z}=\lambda \frac{lv^{2}}{2d},$$

(4) $$h_{j}=\lambda \frac{\tau v^{2}}{2d},\text{ or }h_{j}=\xi \frac{lv^{2}}{2d},$$

where $h_{f}$ denotes the flow resistance; $h_{z}$ and $h_{j}$ represent the straight pipe and local resistance, respectively; ρ is the density of the solution; Δp is the pressure drop; λ and ξ denote the friction coefficient and local resistance coefficient; l and τ denote the length and equivalent length of blood vessels; v is the flow velocity; d is the pipe diameter.

**Figure 4:**
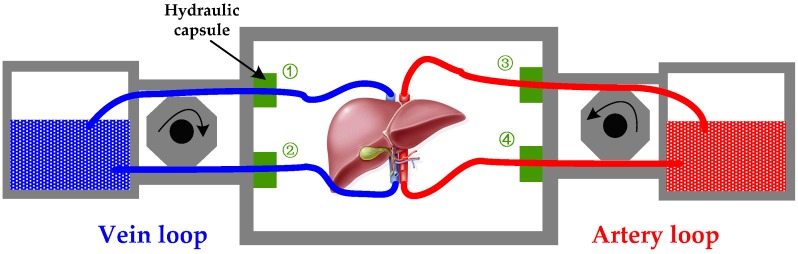
The flow resistance monitoring diagram, which contains a vein loop and an artery loop. The difference between ① and ② means the pressure drop of vein; the difference between ③ and ④ means the pressure drop of artery.

Particularly, the pressure drop Δp is calculated using two hydraulic capsules installed in the artery loop (③ and ④) and the vein loop (① and ②) respectively, which is shown in Figure 4. Then, the flow resistance and other parameters of blood pipe can be obtained according to Equations (2)–(4). Here the measurement range of the hydraulic capsule is 0~50 kPa and the output voltage is 0~10 V. To match the standard AD conversion channels of the TMS320F2812, the output analog voltage will be adjusted to 0~3.3 V using a classical amplifiers, such as an AD623 unit.

Secondly, the solution flow speed is measured by the Hall elements installed in a rotary fan. When the flow liquid pushes the fan blades to run, the distance between the front-end magnet and Hall element will change to generate an impulse current and/or voltage, so the impulse frequency is proportional to monitoring the flow velocity:

(5) v=Vf/s,

where V is the volume between adjacent fan blades; f is the impulse frequency; s is the sectional area of running piping; v is the flow speed gained.

**Figure 5:**
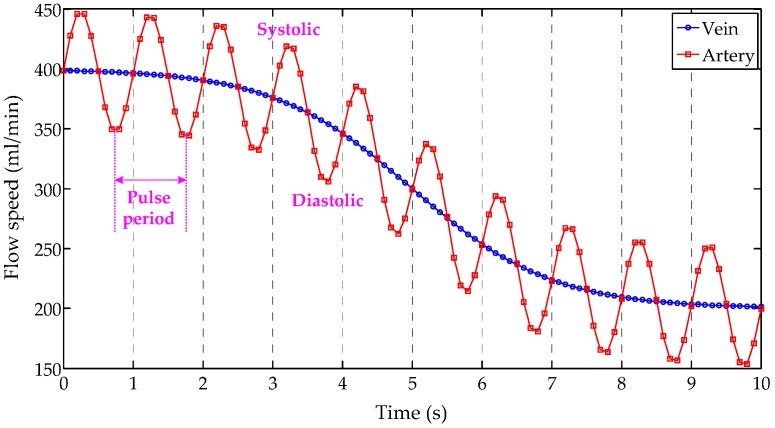
An example of two drive models, (1) vein model: a smooth curve from 400 mL/min to 200 mL/min; (2) artery model: a fluctuating curve from 400 mL/min to 200 mL/min. The peak of artery model reflects the flow speed when the blood is systolic, while the valley reflects the flow speed when the blood is diastolic. Its fluctuating period equals to the heart pulse period.

When we want to increase the energy supply based on the requirements of an organ, the regulation of flow speed is the most efficient and easiest way. As mentioned above, the water-cycle in an HMP system consists of two individual loops: the artery loop and the vein loop. An example of their drive models is shown in Figure 5.

Both two curves display the same process when the flow speed reduces from 400 mL/min to 200 mL/ min. However, they show different dropping styles. The artery loop is run in a pulse way, which simulates the heartbeat process, while the vein loop is carried out in a mild flow way. Beyond that, the comparison between artery and vein loops is listed in Table 2. It can be seen in this table that the flow speed of the arteries is larger than in veins, so, the individual flow speed adjustment can be carried out according to the type of organs and the vein or artery loop in the designed HMP system.

**Figure 6:**
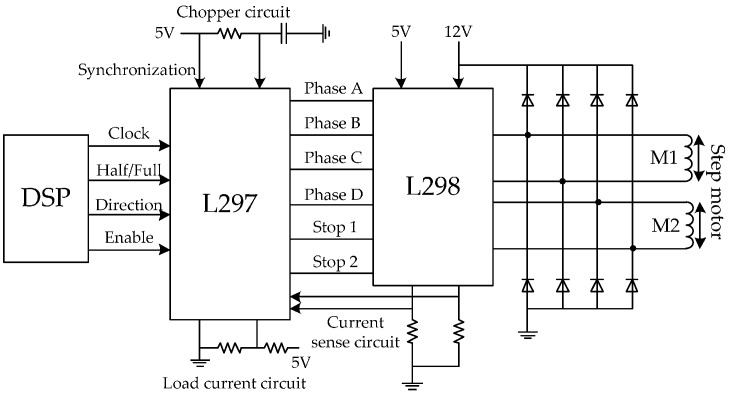
The step motor drive circuit, (1) clock: high clock frequency will cause a faster rotation speed; (2) half/full: the execution efficiency of full-step mode is twice as that of half-step mode; (3) direction: 1/0 represents the positive/negative direction; (4) enable: 1/0 represents controllable/uncontrollable.

In fact, the rotation speed of peristaltic pumps is controlled by two individual step motors in the HMP system, whose drive circuit is drawn in Figure 6. The drive circuit includes the L297 and L298n drive units [24]. The four I/O ports of the F2812 are occupied to control rotating speed, direction, enable and work pattern, respectively. Meanwhile, the input and output of each peristaltic pump are synchronous, which avoids the organ edema (input > output) and lack of energy (input < output). In the drive circuit, the computational formula of flow speed is expressed as:

(6) $$v=\theta_{N}frS,$$

where v is the flow speed of solution; $\theta_{N}$ denotes the single pulse angle of the step motor; f is the pulse frequency; r is the radius of the peristaltic pump; S is the cross-sectional area of the catheter used.

### 3.3. pH Level Monitoring

Generally, the normal concentration of hydrogen ions is equivalent to that of blood in a live organ (arteries: 7.35~7.45, veins: 7.33~7.41). However, the pH level will gradually increase or decrease when a preserved organ begins to degrade, until it loses its activity. In the modified HMP system, a hydrogen ion glass electrode is employed to monitor the pH level and to characterize the deterioration trend of stored organs. The output of this glass electrode meets the Nernst Equation [25]:

(7) $$\Delta E_{M}=\frac{2.303RT}{F}lg\frac{\alpha_{H^{+}}^{2}}{\alpha_{H^{+}}^{1}},$$

where $\Delta E_{M}$ is the potential difference; R is the gas constant; T is the absolute temperature (in degrees Kelvin); F is the Faraday constant; $\alpha_{H^{+}}^{1}$ and $\alpha_{H^{+}}^{2}$ are the hydrogen ion concentration inside and outside the glass electrode, respectively. Although the AD conversion operation is the same as in the flow monitoring, the analog values obtained from glass electrode are weak (20.7 mV~26.6 mV for 7.35~7.45 Hz), so precise signal amplification as well as calibration are required to overcome any interfering noise and compensate the digital output:

(8) $$m_{a}=\left(y_{H}-y_{L}\right)/\left(x_{H}-x_{L}\right),$$

where $x_{L}$ and $x_{H}$ are the low and high input voltages; $y_{L}$ and $y_{H}$ are the low and high output digital signals; $m_{a}$ denotes the actual gain. Then, the gauged gain *C*_1_ and offset *C*_2_ are expressed as:

(9) $$\{\begin{array}{c} \;C_{1}=\left(x_{H}-x_{L}\right)/\left(y_{H}-y_{L}\right)\;\\ \;C_{2}=y_{L}/m_{a}-x_{L} \end{array},$$

Then, the ADC process in the F2812 is carried out as follows:

- Setting the interrupt vector table of F2812: point the INT1.6 vector to the ADC module, which will be triggered if the AD sign is free;
- Allocate the registers: (1) sample mode: start/stop and cascade mode; (2) conversion mode: synchronous; (3) maximal conversion number: 15; (4) trigger mode: internal;
- Enter the ADC interruption sub program of hydraulic pressure or pH level monitoring:
  (10) $$A_{x}=3C_{1}\left(D_{x}+C_{2}\right)/4095,$$
  where $A_{x}$ is the analogue output of $D_{x}$. Finally, the hydraulic pressure and pH value by restoring the amplification of amplifiers:
  (11) $$\{\begin{array}{c} \;H=500\times \left(\bar{A_{\mathrm{Pr}1}}-\bar{A_{\mathrm{Pr}2}}\right)/3\rho \\ \;pH=14\times \bar{A_{\mathrm{pH}}}/3 \end{array},$$
  where $\bar{A_{\mathrm{Pr}1}}$, $\bar{A_{\mathrm{Pr}2}}$ and $\bar{A_{\mathrm{pH}}}$ are the mean of $A_{\mathrm{pH}}$, $A_{\mathrm{Pr}1}$ and $A_{\mathrm{Pr}2}$ of each five times, which reduces the random error at the cost of reducing the sampling frequency.

## 4. Thermostatic Control of the HMP System

Generally, there are two main measures to improve the reliability of temperature regulation: (1) Multi-intelligent algorithms, such as the neural network [26], predictive control [27], Proportion Integration Differentiation (PID) controller [28] and genetic algorithm [29], are combined to enhance the response sensibility, but this adds complexity and computational load and is thus inapplicable in a real-time monitoring device. (2) Reduce the inertia or time-lag between the measured piece and sensors by adding a compensation unit. For instance, the capability of temperature tracking and the linearity of output were heightened by bringing in an adaptive compensation algorithm by Chotai and Young [30]. However, the application to related applications, such as the measurement range, is limited. In this paper, a thermostatic control strategy based on multi-sensor data fusion and a fuzzy-PID method is proposed to improve the temperature control accuracy aiming at the HMP research object.

### 4.1. Equipment and Materials

**Figure 7:**
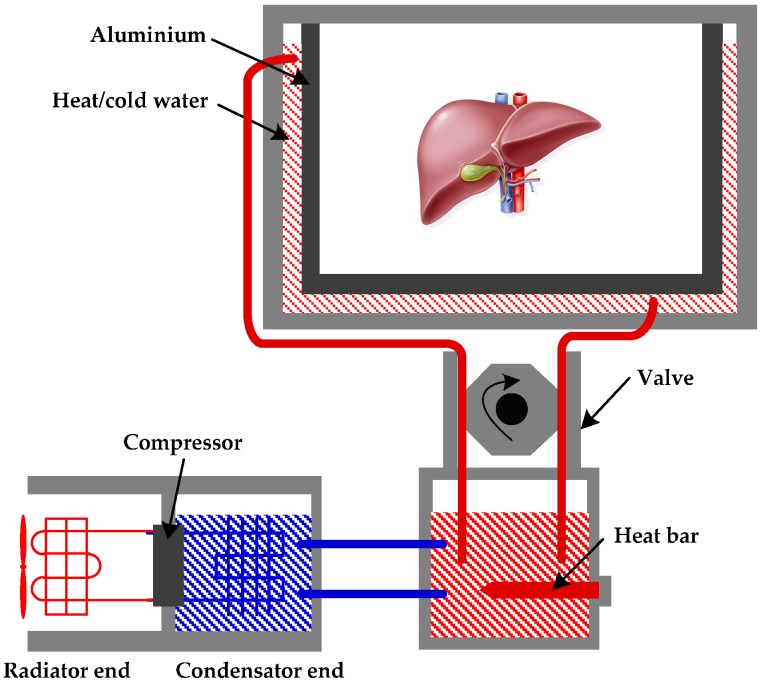
The thermostatic control equipment, i.e., a compressor and a heat bar, are controlled by DSP to increase and reduce the temperature of water, respectively. After a controllable valve, the water can reach the thin aluminium alloy around the stored organ. After that, the heat of water can penetrate the metallic material and adjust the organ temperature.

The main equipment and materials involved in the temperature control process include a compressor, a heat bar and a valve, as indicated in Figure 7.

Compressor: the refrigeration capacity of the compressor can be calculated as follows [31]:

(12) $$Q_{c}=\frac{vnq_{v}}{3.6\;\times \;10^{6}}\left(\mathrm{kW}/h\right),$$

where $Q_{c}$ denotes the refrigerating capacity; $q_{v}$ is the unit volume refrigerating capacity; v is the displacement per rotate of compressor; n is the rotational speed of compressor. When we take the energy loss into consideration, the refrigeration quantity of the organ gain is:

(13) $$Q_{c}^{\prime }=\left(1-\xi \right)\times \frac{v_{1}}{v_{m}}\times \frac{vnq_{v}}{3.6\times 10^{6}}\left(\mathrm{kW}/h\right),$$

where ξ denotes the energy loss efficiency in the transfer pipeline; $v_{1}$ is the rotation speed of the valve; $v_{m}$ denotes the maximal rotation speed of the valve.

Heat bar: the heating capacity of the heat bar can be calculated using Equation (14):

(14) $$Q_{h}=\frac{\eta Q_{e}}{3.6\times 10^{6}}\left(\mathrm{kW}/h\right),$$

where $Q_{h}$ denotes the heating capacity; $Q_{e}$ is the electric energy that the heat bar consumes; η denotes the conversion efficiency.

Therefore the heating quantity of the organ gain is:

(15) $$Q_{h}^{\prime }=\left(1-\xi \right)\times \frac{v_{1}}{v_{m}}\times \frac{\eta Q_{e}}{3.6\times 10^{6}}\left(\mathrm{kW}/h\right),$$

The TXD/RXD ports of the DSP are especially designed to communicate with the drive circuit of the compressor with a 600 bps baud rate. When a target rotation speed is needed, the DSP will send the value to the compressor, while the current states of the compressor will also be returned to the microcontroller. The data format of the communications between the DSP and compressor is shown in Table 3.

### 4.2. Data Fusion of Temperature Sensors

With increasing attention to the stability of products, the multi-sensor data fusion technique based on the Bayes method [32] has been developed. This technology, targeting data from multiple sensors, can achieve better accuracy than a single sensor alone and has been applied in various areas such as target tracking [33], automated identification [34], and also in the HMP system. To be specific, the input of the algorithm is the vector $Z=\left\{z_{1},z_{2},z_{3},z_{4}\right\}$, whose elements represent the digital values of TSic506 units, and the output is the fused temperature T={t}. In the classical Bayes estimation, the computational formula of posterior probability distribution is given by Equation (16):

(16) $$p\left(t_{k}|Z^{k}\right)=\frac{p\left(z_{k}|t_{k}\right)p\left(t_{k-1}|Z^{k-1}\right)}{p\left(Z^{k}|Z^{k-1}\right)},$$

where $p\left(z_{k}|t_{k}\right)$ is the likelihood function which depends on the sensor; $p\left(t_{k}|Z^{k}\right)$ is the prior distribution function which relates to the transformation model; $p\left(Z^{k}|Z^{k-1}\right)$ is the normalized function of probability density; k and k−1 represent the current and last moment.

It is noted that the four observed temperature values are only the approximation of real temperature due to the indeterminacy of sensors and the interfering noise. This can be described using the Gaussian distribution in Equation (17):

(17) $$p\left(Z=z_{j}|T=t\right)=\frac{1}{\sigma_{j}\sqrt{2\pi }}exp\left\{\frac{-{\left(t-z_{j}\right)}^{2}}{2\sigma_{j}^{2}}\right\},$$

where $\sigma_{j}$ represents the variance of the jth sensor, j∈ {1, 2, 3, 4}. Finally, the fused vector T={t} can be calculated according to the maximum a posteriori equation:

(18) $$t=\frac{\sigma_{1}^{-2}}{\delta }z_{1}+\frac{\sigma_{2}^{-2}}{\delta }z_{2}+\frac{\sigma_{3}^{-2}}{\delta }z_{3}+\frac{\sigma_{4}^{-2}}{\delta }z_{4},$$

where δ =$\;\sigma_{1}^{-2}\;$+$\;\sigma_{2}^{-2}\;$+$\;\sigma_{3}^{-2}\;$+$\;\sigma_{4}^{-2}$ and the variance of fused temperature $\sigma_{t}^{2}$ = 1/δ.

Unfortunately, error messages, resulting from faulty sensors or interfering noise, may lead to the failure of fusion. The traditional Bayes estimation will lose efficacy due to the average weight allocation to all channel data. A modified method, which introduces an improvement factor, is used aiming to reducethe possible data errors. The improved Gauss formula is expressed in (19):

(19) $$p\left(Z=z_{j}|T=t\right)=\frac{1}{\sigma_{j}\sqrt{2\pi }}exp\left\{\frac{-{\left(t-z_{j}\right)}^{2}}{2\sigma_{j}^{2}f}\right\},$$

where the improvement factor f is identified as:

(20) $$f=\frac{m^{2}}{m^{2}-{\left(max\left(z_{j}^{k}-z_{j}^{k-1}\right)\right)}^{2}},$$

where m is the acceptable maximum deviation of the TSic506 unit; $z_{j}^{k}$ and $z_{j}^{k-1}$ are the current and the last observed values, respectively. The difference between $z_{j}^{k}$ and $z_{j}^{k-1}$ is positively associated with the probability of the fault. The relevant improvement factor f will increase once an error occurs in a certain temperature datapoint. Then its effect will be weakened by adjusting its variance in (19). In consequence, the error messages in obtained data can be recognized and eliminated.

**Figure 8:**
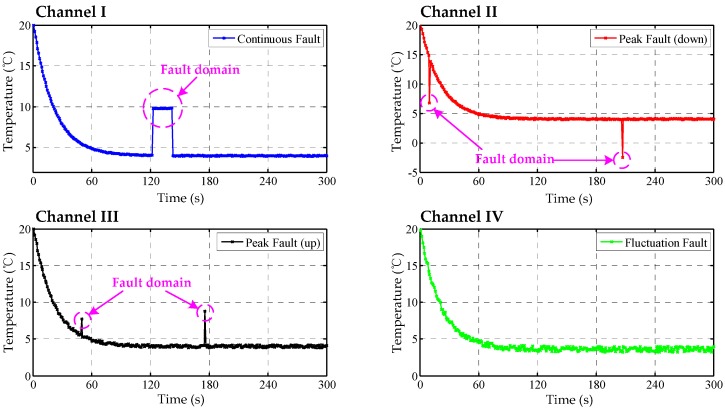
The data curves of TSic506 units, channel I: continuous fault resulting from sensor fault; channel II: down peak fault resulting from accidental fault; channel III: up peak fault resulting from accidental fault; channel IV: fluctuation fault resulting from interfering noise.

For verifying the suitability of the data fusion approach, four temperature datasets were analyzed, which considered possible fault factors. The sampling frequency and time were set as 1 Hz and 300 s, respectively. As shown in Figure 8, the temperature trends of the four channels are all from 20 °C to 5 °C, and contain examples of a continuous fault, peak faults and a fluctuation fault.

**Figure 9:**
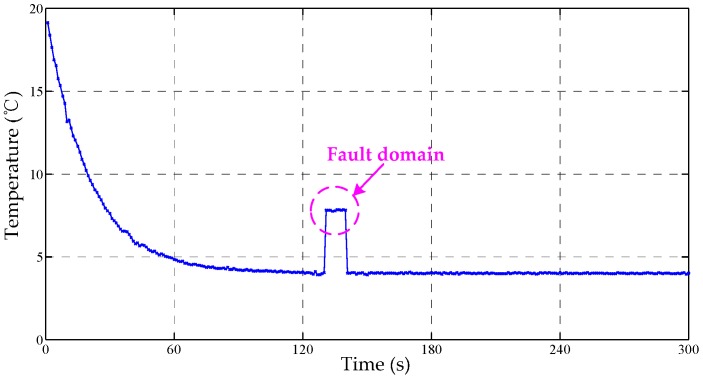
The fused Bayes estimation curve.

**Figure 10:**
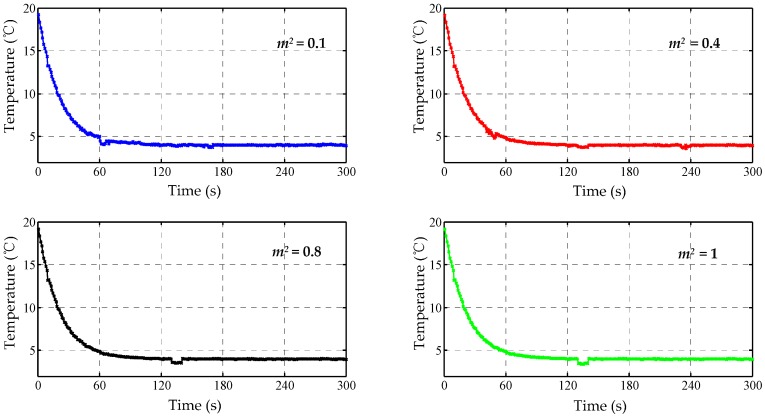
The fused curves of the modified Bayes estimation.

These temperature data were divided into 30 stages and were fused using the classical/modified Bayes estimation methods. Test results are drawn in Figure 9 and Figure 10. For the modified method, the acceptable maximum differences in Equation (20) were set as the next four levels: $m^{2}\;$= 0.1, 0.4, 0.8, 1.

From Figure 9, the peak error of the Bayes curve is effectively suppressed. Meanwhile, the fluctuation or the variance of error is reduced by 34.3% compared to the original curve in Channel IV. By contrast, the continuous error, resulting from a possible faulty sensor, still exists in the fused curve, which indicates the limitations of the traditional method. From Figure 10, it is found that the small value m is beneficial to the correction of the continuous error, while a large m value assists to identify the peak fault. Therefore, the acceptable maximum deviation ($m^{2}\;$ = 0.4) can decrease both errors greatly. Particularly, the decreased error in Figure 10 indicates that the fusion performance of the modified method is preferable to the traditional strategy.

**Figure 11:**
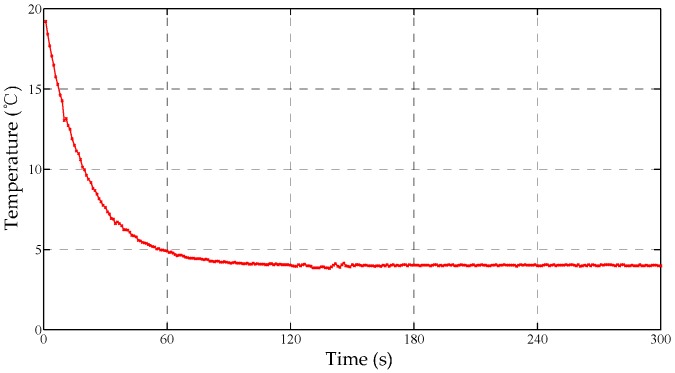
The fused curve of the cascaded Bayes estimation.

Furthermore, the cascaded Bayes estimation is proposed to overcome the randomness of the maximum difference *m* in the modified Bayes estimation. Based on the results in Figure 10, four fused curves are regarded as the new input of the second order Bayes estimation. The cascaded performance, seen from Figure 11, is improved compared to the first order. 

### 4.3. Thermal Control Based on Fuzzy-PID

**Figure 12:**
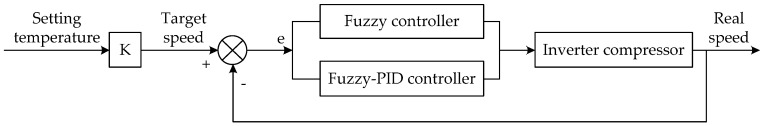
The schematic diagram of thermal control. If the temperature deviation is large, the single fuzzy controller will be adopted to improve the response speed and reduce the negative overshoot; Otherwise, a fuzzy-PID controller will be carried out to improve the accuracy.

The direct thermal control method is to accurately control the inverter compressor. Then the DSP predicts the temperature in measuring points according to Equation (21):

(21) $$T_{n}=T_{c}+\frac{vnq_{v}}{c_{0}m_{0}+c_{1}m_{1}},$$

where $T_{n}$ and $T_{c}$ represent the predicted and current temperature, respectively; $c_{0}$ and $c_{1}$ are the specific heat of air and the solution; $m_{0}$ and $m_{1}$ are the air and solution mass inside the organ box; $q_{v}$ is the unit volume refrigeration capacity; v is the gas displacement of the compressor per rotation; n is the rotational speed. Then, the basic idea of thermal control is drawn in the schematic diagram of Figure 12.

#### 4.3.1. Fuzzy Controller

**Figure 13:**
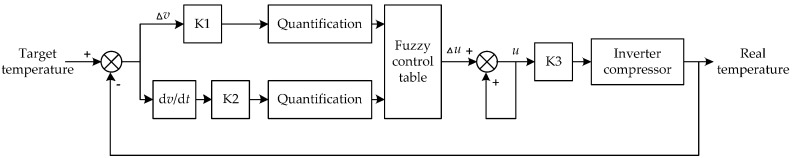
The schematic diagram of fuzzy control. Here ∆*u* is the control quantity; ∆*v* is the motor speed; *cv* = d*v*⁄d*t* is the change rate of speed; K1 & K2 are the quantification factors; K3 is the scale factor. Where ∆*u*, ∆*v*, ∆*cv* are three domains of discourse of fuzzy controller.

The essence of a fuzzy controller [35] is to map the measured temperature to a fuzzy subset, which contains a series of linguistic terms, using the fuzzy inference. The general process is described as four steps: fuzzification, calling the repository, fuzzy inference and defuzzification. Figure 13 indicates that the control quantity of compressor can be obtained by using the motor speed and its change-rate as inputs, and uses an off-line fuzzy control table as mapping criterion.

**Figure 14:**
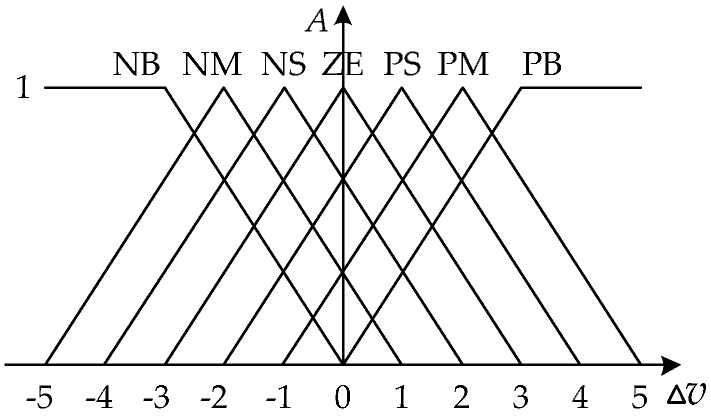
The membership function A(X)∈ [0, 1] (X∈ {Δu, Δv, cv}), the higher value indicates the more possibility that X is classified into a certain related segment.

The details of fuzzy controller are listed in the next three steps:

- Segment different levels of temperature deviation and define the membership function. ∆*u*, ∆*v* and ∆*cv* are divided into seven segments as {−3, −2, −1, 0, 1, 2, 3}, which represent negative-big (NB), negative-middle (NM), negative-small (NS), zero (ZE), positive-small (PS), positive-middle (PM) and positive-big (PB) levels. The membership function A(X) is shown in Figure 14.
- Design the fuzzy rule: “If ∆*v* and *cv*, then ∆*u*”, which describes the relationship between the input and the output. For example, if ∆*v* and *cv* are both NB level, which means the real speed is away from the setting value and the variation trend even lets the result worse, so ∆*u* should be set as NB level to reduce the motor output. More fuzzy rules are listed in Table 4.
- Defuzzification. The fuzzy reference list is also given in Table 4 by calculating the membership function based on the maximum membership criterion.

#### 4.3.2. Fuzzy-PID Controller

Actually, it is hard to eliminate the stable errors in a single fuzzy controller because of its fuzziness. An accurate mathematic model, named Fuzzy-PID controller [36], is designed to weaken the fluctuation by combining the fuzzy controller with PID controller when the temperature deviation is small. The discrete formulation of PID is expressed as:

(22) $$u\left(k\right)=k_{p}e\left(k\right)+k_{i}\sum_{j\;=\;0}^{k}e\left(j\right)T+k_{d}\frac{e\left(k\right)\;-\;e\left(k\;-\;1\right)}{T},$$

where u(k) is the output quantity; e(k) is the deviation of measured and setting temperature; e(k−1) is the deviation at last moment; T is the sampling period; $k_{p}$, $k_{i}$ and $k_{d}$ denote the proportion, integral and derivative coefficients, respectively.

**Figure 15:**
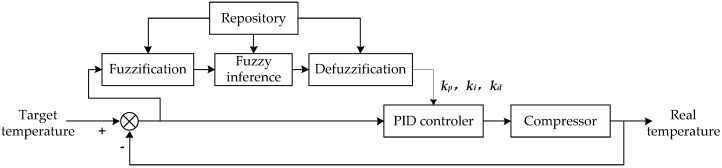
The flow diagram of fuzzy-PID method. Here three parameters $k_{p}$, $k_{i}$ and $k_{d}$ are adjusted using the fuzzy controller, then they are set as the proportion, integral and derivative coefficients of the PID controller.

However, it is difficult to obtain the exact $k_{p}$, $k_{i}$ and $k_{d}$ value with experience, so the fuzzy controller is utilized to optimize these three parameters in a fuzzy-PID algorithm, whose flow diagram is shown in Figure 15.

During the process, the input variable ΔT and the output variables $k_{p}$, $k_{i}$ and $k_{d}$ are divided into five segments {NM, NS, ZE, PS, PM}. After fuzzy computing, the fuzzy rules and references are listed in Table 5. It can be seen that the proportion and integral coefficients go down mildly while the derivative coefficient goes up from 0 slightly when the temperature-deviation starts from negative to positive. In addition, the arithmetic speed of combined method, proved by extra experiments, is faster than single PID algorithm.

## 5. Experiments and Performance Analysis

**Figure 16:**
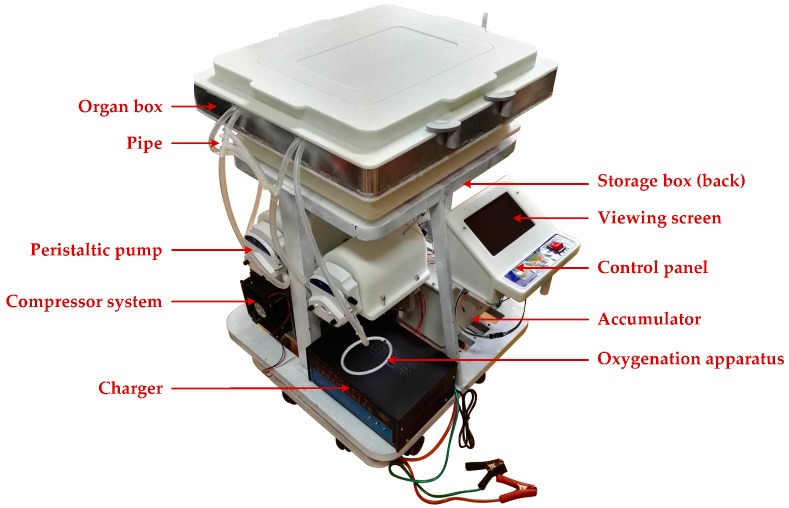
The hypothermic machine perfusion (HMP) device.

Figure 16 illustrates the physical view of the designed HMP system according to the modified HMP framework in Figure 1b. Some tests have been performed to verify its reliability, listed in following sub-sections.

### 5.1. Dynamic Thermostatic Control Properties

**Figure 17:**
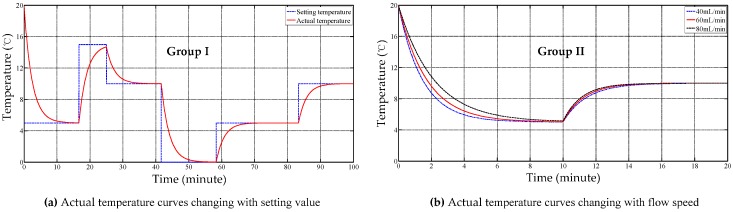
The dynamic performance trial curves.

Two tests, illustrated in Figure 17, were carried out to verify the dynamic properties of the proposed approaches which use water as the perfusion medium in the HMP system. Group I: the target temperatures were set as 5, 15, 10, 5, 0 °C at the 16.7, 25, 41.7, 58.3 and 83.3 min moment, respectively. The flow speed was set as 40 mL/min; Group II: the target temperature was set as 5 and 10 °C and the flow speeds were set as 40, 60 and 80 mL/min.

From Figure 17a, the initial real temperature gradient is positively associated with the deviation. The maximal gradient is 3.4 °C/min when the fuzzy control motor speed variation is set as PB or NB; From Figure 17b, the time-costs of 15–10 °C and 10–5 °C are 1.2 min and 6.2 min when the target temperature is set as 5 °C, which is associated with the proportional and differential coefficients. Meanwhile, a fast flow speed leads to a slow temperature dropping tendency, which depends on the effect of external interference on the flowing solution.

**Figure 18:**
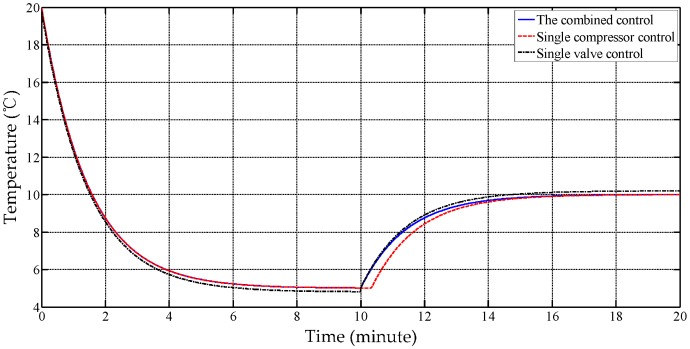
The trial curves of different working modes.

In addition, we compared three working modes, including the single compressor mode, the single valve mode as well as the combined mode, and the temperature curves of different modes are drawn in Figure 18. The figure shows that the compressor response is slower than that of the valve due to the curving pipes and the compressor delay. However, the stability of the former is better than the latter because of the compressor’s continuously adjustable rotation speed. Therefore, the combination is of benefit to balance the sensitivity and the accuracy performance.

### 5.2. Static Property of Thermostatic Control

Generally, the accuracy of temperature control is concerned with the integration coefficient $k_{i}$ directly. For testing the static stability, the standard deviations of the error for flow speeds under the conditions of 40, 60 and 80 mL/min are listed in Table 6. It indicates that the static performance depends on the flow situation rather than the target temperature. To be specific, the average standard deviation in 40 mL/min decreases 53.49% compared to the 80 mL/min flow speed. However, the high flow speed helps to wash away the metabolic waste and prevent organ deterioration.

The experiment also analyzed the static performance of three working modes, listed in Table 7. It is observed that the accuracy is ranked in the order combined mode > single compressor mode > single valve mode. A slight fluctuation exists in single valve control due to its open duty cycle design being limited to 0.8%. However, the combined mode improves the accuracy at the cost of the energy use-ratio. For example, the 50% open duty cycle means half the available energy is wasted.

### 5.3. Other HMP Performance Analysis

During other performance analysis, the EC solution, the UW solution and the Celsior solution were taken into consideration to be the perfusion solutions, respectively. The function tests, including the sensor tests, the LCD tests and the perfusion tests, indicated that the system is appropriate for isolated organs. Also, two oxygenation apparatus are installed in the HMP system to provide the oxygen for saving organs, in the artery and vein system respectively. In addition, the durability of the instrument was testified to meet the design requirements and the system should be of potential value to the modern medical field.

Finally, why is this device a kind of low-cost system compared with traditional HMP device? There are three reasons: (1) the wide applicability of our equipment to different organs. Currently, the physical parameters of most HMP devices are designed for a certain organ, such as the LifeCradleTM system for hearts, the Groningen Machine Perfusion for livers, the LifePort system for kidneys and pancreas. On the contrary, the temperature and flow rate are controllable in our device, thus meeting the different requirements of all kinds of organs and saving the cost of replacing devices. (2) Using the artificial lung, the perfusion solution is recyclable and self-renewing in the designed system, which saves the cost of replacing solutions. (3) Monitoring of physical parameters is automatic, which saves the cost of maintenance in traditional manual manipulation devices.

## 6. Conclusions

A hypothermic machine perfusion system is realized in this paper to maintain a suitable organ living surrounding and extend the survival life of isolated organs. Four elements, including a machine perfusion system, sensor monitoring, thermostatic control and an oxygenation apparatus, are involved in this system. In particular, we improve the replacement efficiency of different organs by controlling the physical parameters. The result proves the satisfactory monitoring performance in real time of the degradation trend or current states of stored organs.

In addition, a two-order modified Bayes estimation, which introduces the concept of an improvement factor, is realized to weaken the possible temperature errors resulting from sensor faults and noise interference. Compared with the traditional PID controller, the accuracy is improved and the computation cost is low. Experiments also proved that the dynamic properties are concerned with the external temperature, while the static properties mainly depend to some degree on the flow conditions.

## Figures and Tables

**Table 1:** A comparison between the three strategies for kidney perfusion [8,9,10,11].

Feature	SCS	NMP	HMP
The average preservation time	9.5 h	12 h	13 h
Organ survival rate after 15 months	85.0%	87.7%	89.2%
Organ survival rate after 5 years	54.2%	68.7%	70.5%
Facility cost	inexpensive	expensive	expensive

**Table 2:** A comparison between artery and vein loops.

	Pipe Wall	Lumen	Flow Speed
**Artery**	Thick and elastic	Rather small	Rather fast
**Vein**	Rather thick and weak elastic	Rather big	Rather slow

**Table 3:** The data format between DSP and compressor.

**TXD (0x)**	1	2	3–4		5–13	14
	Address	On/Off	Speed		00	Check
**RXD (0x)**	1	2–3	4–5	6	7–13	14
	Address	Speed	Voltage	Fault	00	Check

**Table 4:** The lists of fuzzy rules (∆*u*)/references.

	Δv	NB/−3	NM/−2	NS/−1	ZE/0	PS/1	PM/2	PB/3
cv								
**NB**		NB/3	NB/3	NM/2	NM/2	NS/1	NS/1	ZE/0
**NM**		NB/3	NM/2	NM/2	NS/1	NS/1	ZE/0	PS/−1
**NS**		NM/2	NM/2	NS/1	NS/1	ZE/0	PS/−1	PS/−1
**ZE**		NM/2	NS/1	NS/1	ZE/0	PS/−1	PS/−1	PM/−2
**PS**		NS/1	NS/1	ZE/0	PS/−1	PS/−1	PM/−2	PM/−2
**PM**		NS/1	ZE/0	PS/−1	PS/−1	PM/−2	PM/−2	PB/−3
**PB**		ZE/0	PS/−1	PS/−1	PM/−2	PM/−2	PB/−3	PB/−3

**Table 5:** The lists of fuzzy rules/ references.

Input	ΔT	NM/−2	NS/−1	ZE/0	PS/1	PM/2
**Output**	$k_{p}$	PM/0.03	PS/0.025	ZE/0.02	NS/0.015	NM/0.01
	$k_{i}$	PM/0.035	PS/0.031	ZE/0.027	NS/0.023	NM/0.02
	$k_{d}$	NM/0	NM/0	NS/0.005	ZE/0.010	PM/0.02

**Table 6:** The standard deviations of error.

Flow Speed (mL/min)	Setting Temperature (℃)	Maximum Temperature (℃)	Minimum Temperature (℃)	Standard Deviation (℃)
40	+5	+5.17	+4.85	0.1091
	+15	+15.19	+14.82	0.1056
60	+5	+5.29	+4.77	0.1613
	+15	+15.25	+14.72	0.1642
80	+5	+5.41	+4.66	0.2364
	+15	+15.38	+14.57	0.2348

**Table 7:** The static performance of different modes.

Mode	Setting Temperature (℃)	Standard Deviation (℃)	Power Consumption
Single compressor	+5	0.8331	Minimum
	+15	0.6517	
Single valve	+5	0.4920	Maximum
	+15	0.4501	
Combination	+5	0.0813	Middle
	+15	0.1073	
